# Consequences of hemolytic uremic syndrome among hemodialysis patients

**DOI:** 10.1007/s40620-014-0149-x

**Published:** 2014-12-10

**Authors:** Steven M. Brunelli, Ami Claxton, Sunil Mehta, Emmanuel A. Anum

**Affiliations:** 1 DaVita Clinical Research, 825 So. 8th St, Minneapolis, MN 55404 USA; 2 Alexion Pharmaceuticals, Cheshire, CT USA

**Keywords:** Hospitalization, Mortality, Survival

## Abstract

**Background:**

Hemolytic uremic syndrome (HUS) is characterized by hemolytic anemia, low platelets, and renal impairment and is mediated by thrombotic microangiopathy (TMA). A common perception is that HUS becomes dormant in dialysis patients with end-stage renal disease (ESRD). We analyzed patients in a large dialysis organization to understand the potential consequences and burden of HUS.

**Methods:**

We identified patients with ESRD ascribed to HUS and those with ESRD ascribed to another cause (control patients) who received hemodialysis or peritoneal dialysis from 01 January 2007 to 31 December 2012. Outcomes were survival, hospitalization, and longitudinal laboratory values associated with TMA, including lactate dehydrogenase, red cell distribution width (RDW), platelets, and hemoglobin.

**Results:**

HUS patients (n = 217) were propensity-score matched 1:5 to control patients (n = 1,085) for age, gender, race, dry weight, insurance, access, comorbidities, and Charlson comorbidity index. Compared to control patients, HUS patients had significantly greater risk for hospitalizations overall (RR = 2.3, p = 0.004) and hospitalization for hematologic (RR = 5.6, p = 0.001), cardiovascular (RR = 2.1, p = 0.02), and pancreatic (RR = 7.9, p = 0.04) causes. HUS patients also had evidence of ongoing TMA: higher lactate dehydrogenase and RDW, lower platelets and hemoglobin, and more frequent lactate dehydrogenase spikes.

**Conclusions:**

Dialysis patients with HUS were at significantly higher risk than matched control patients for hospitalizations due to cardiovascular, hematologic, and pancreatic disease, which were associated with ongoing TMA. Additional studies are needed to determine whether targeted therapy for HUS reduces hospitalizations.

## Introduction

Hemolytic uremic syndrome (HUS) is a devastating disease that is mediated by thrombotic microangiopathy (TMA). Historically, patients with the disease present with a triad of clinical signs: thrombocytopenia, hemolytic anemia, and acute renal failure [1, 2]. It is increasingly recognized that in addition to renal and hematologic injury, TMA affects nearly every organ system, including (but not limited to) the central nervous, cardiovascular, and digestive systems [1].

There are two types of HUS, typical and atypical. Typical HUS is bacterial in origin, accounts for 90 % of HUS patients, and generally does not lead to renal failure in adults [3–6]. Atypical HUS is a genetic disease in which excessive complement activity leads to TMA, hemolytic anemia, and acute renal failure [5, 7–9]; it is estimated that 64–67 % of adults with atypical HUS die or reach end-stage renal disease (ESRD) within 3–5 years of onset [10].

A common perception among clinicians is that HUS becomes dormant following progression to ESRD. This perception may stem, in part, from the inability of patients to manifest further renal injury in the context of renal failure. However, emerging evidence indicates that HUS patients continue to manifest signs and symptoms of TMA after the onset of ESRD. For example, a 2006 study by Perkins et al. [11] found that the rate of overt TMA in dialysis patients with HUS-ascribed ESRD was 11.3 % in the first year of dialysis and remained at about 4.5 % every year thereafter; the TMA rate among dialysis patients without HUS averaged about 0.3 % per year. The researchers also found that TMA was independently associated with an increased risk of death in the first year following a TMA diagnosis.

At present, it remains unknown whether morbidity and mortality differ between patients with ESRD due to HUS (which disproportionally consists of atypical versus shiga toxin-related disease), versus comparable patients with ESRD due to other etiologies. To clarify burden of disease, we compared survival, hospitalization, cause-specific hospitalization, and longitudinal laboratory patterns between patients with ESRD ascribed to HUS versus propensity-matched control patients with ESRD ascribed to a cause other than HUS or TMA-related conditions.

## Subjects and methods

### Patients

We conducted a retrospective study of adult ESRD patients from a large dialysis organization (LDO) who began maintenance in-center hemodialysis or peritoneal dialysis between 01 January 2007 and 31 December 2012. Demographic and laboratory data were obtained from the LDO’s clinical data warehouse, which stores the electronic health records. Hospitalization events and cause-attribution data [based on International Classification of Diseases, Ninth Revision (ICD-9) codes] were obtained from Medicare Claims files, which are made available through the United States Renal Data System (USRDS) and linked to the LDO’s electronic health records. Hospitalization analyses were limited to Medicare patients and were considered from 01 January 2007 through 31 December 2010 (the last date of available claims data).

**Fig. 1:**
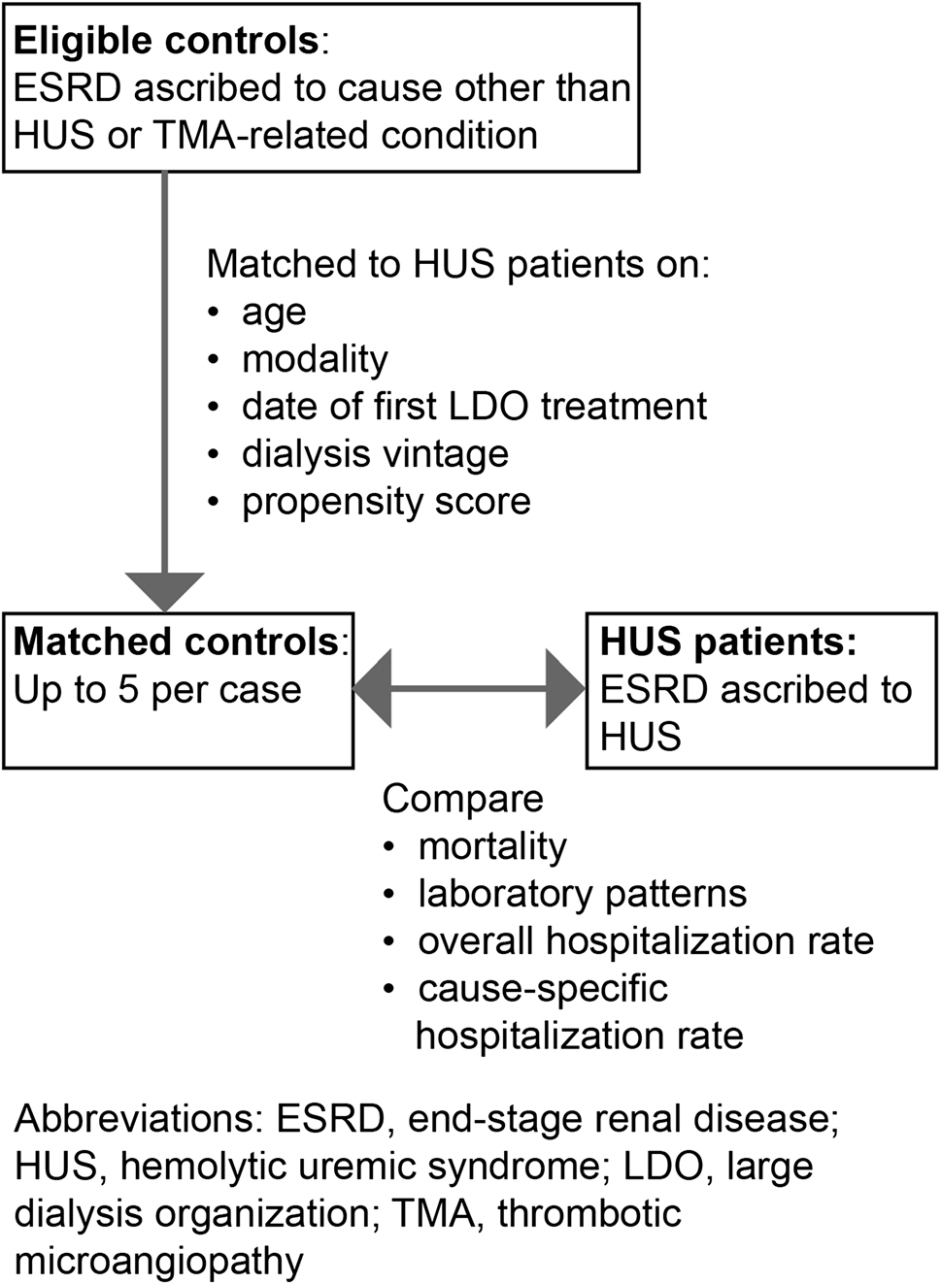
Identification of study patients. Identification of patients with end-stage renal disease (ESRD) ascribed to hemolytic urinary syndrome (HUS) and matched control patients with ESRD ascribed to neither HUS nor a thrombotic microangiopathy (TMA)-related condition. TMA-related conditions are defined as lupus, scleroderma, antiphospholipid antibody syndrome, malignant hypertension, eclampsia/preeclampsia/post-partum renal failure, cholesterol emboli syndrome, Budd–Chiari syndrome, paroxysmal nocturnal hemoglobinuria, acute interstitial nephritis, HIV-associated nephropathy, and heroin nephropathy

The HUS patients were identified as incident ESRD patients who began dialysis at the LDO during the study period with ESRD ascribed to ICD-9 code 283.11 (hemolytic uremic syndrome) (Fig. 1). As there is no ICD-9 code specific for *atypical* HUS, both atypical and diarrheal-associated disease were considered together. Because most typical cases of HUS occur in children <4 years of age [12] and because consideration in this study was limited to adult patients, we presumed the majority of HUS patients had atypical disease. Eligible control patients were adult patients who began dialysis at the LDO during the study period with ESRD ascribed to any etiology other than HUS or a TMA-related condition (i.e., lupus, scleroderma, antiphospholipid antibody syndrome, malignant hypertension, eclampsia/preeclampsia/post-partum renal failure, cholesterol emboli syndrome, Budd–Chiari syndrome, paroxysmal nocturnal hemoglobinuria, acute interstitial nephritis, human immunodeficiency virus (HIV)-associated nephropathy, and heroin nephropathy).

The HUS patients were propensity score matched [13, 14] (1: many; with ratio up to 5) without replacement to eligible control patients on the basis of age, sex, race/ethnicity, primary insurance provider, body weight, dialysis vintage, dialytic modality, and the presence (at study entry) of hypertension, coronary disease, congestive heart failure, cerebrovascular disease, and peripheral vascular disease. Maximum caliper width was set to <0.0001.

### Measurements

Patients were considered at risk beginning at the time of dialysis initiation and continuing until death or kidney transplant, transfer of care away from the LDO, recovery of renal function, or end-of-study period (31 December 2012; 31 December 2010 for hospitalization analyses).

Baseline characteristics of HUS patients were described as means, standard deviations (SDs), medians, interquartile ranges, counts, and proportions as dictated by data type (continuous or noncontinuous variables). These baseline characteristics were compared between groups using standardized differences; standardized differences of absolute value <10 % are indicative of good balance between groups.

### Statistical analysis

Survival was compared using Kaplan–Meier analysis, log rank testing, and Cox proportional hazards regression; regression models were stratified on matched group to account for the matched design. The proportionality assumption was assessed by fitting models with 2-way exposure-by-time cross-product terms. Statistical significance of the cross-product term would have been interpreted as evidence of non-proportionality; this was not observed, suggesting that associations were time-invariant.

Rates of hospitalization overall and for each cause-specific type were compared between HUS patients and control patients using generalized linear models. Models were specified with a log link and Poisson distribution and contained random effects intercepts for patients and fixed effects terms for exposure status and time. A variance component matrix was assumed when controlling for patient correlation (longitudinal observations) over time.

Data on laboratory indices of TMA—platelet count, lactate dehydrogenase, hemoglobin, and red cell distribution width (RDW)—were extracted from the LDO’s electronic health record. These parameters were compared longitudinally between study groups using linear mixed models. Missingness was assessed by creating a series of missing indicator variables (=1 if missing; =0 if present). For each variable, the association of missingness with exposure was then assessed using a mixed linear model with a logit link and binomial distribution, with random effects intercept for patient and fixed effects terms for exposure and time (the latter to account for secular patterns of missingness).

Significant associations between HUS/control status and missingness would have been interpreted as evidence of violation of the missing-at-random assumption; this was not observed for any variables (p > 0.05 for each).

Longitudinal lactate dehydrogenase spikes were considered as monthly longitudinal dehydrogenase values that were 100 U/L or more greater than the patient’s mean value over the prior 2 months; these were compared between HUS patients and control patients in an analogous manner. Analyses of the association between lactate dehydrogenase spikes and hospitalizations involved a dichotomous response variable (hospitalization 0/1 in the response month) and a time-varying dichotomous exposure variable (lactate dehydrogenase spike 0/1 from prior month). These associations were therefore analyzed using a time-updated linear mixed model with a logit link (to account for the dichotomous nature of the outcome) and estimates.

## Results

Each HUS patient (N = 217) was successfully matched to 5 control patients (N = 1,085) from a pool of 230,668 eligible subject patients (Table 1). Unlike in the source cohort, HUS patients and control patients in the matched population were well balanced on covariates: both groups averaged 48 years and were 57 % female. Race distribution was similar (75 % white for HUS patients, 74 % for control patients, and 16 % black patients for both case and control groups). Medicare was the primary source of insurance for 35 % of both HUS patients and control patients. Regarding vascular access, 82 % of HUS patients versus 81 % of control patients had central venous catheter access. For HUS patients versus control patients, comorbidity patterns were similar for diabetes (11 versus 10 %), hypertension (47 versus 44 %), coronary artery disease (5 versus 4 %), congestive heart failure (9 versus 8 %), cerebrovascular disease (3 % for both groups), and peripheral artery disease (2 % for both groups).

**Table 1:** Comparison of baseline characteristics between hemolytic uremic syndrome patients and source and matched cohorts

Variable	HUS patients	Source cohort		Matched cohort	
	n = 217	n = 230,668	Std diff	n = 1,085	Std diff
Age (years), mean ± SD	48 ± 18	63 ± 15	−1.0	48 ± 16	0.02
Sex, n (%)					
Male	93 (43 %)	97,921 (42 %)	−0.3	467 (43 %)	0.0
Female	124 (57 %)	132,681 (58 %)		618 (57 %)	
Race, n (%)					
White	163 (75 %)	116,119 (50 %)	0.5	803 (74 %)	0.1
Black	35 (16 %)	66,812 (29 %)		170 (16 %)	
Other	19 (9 %)	47,472 (21 %)		112 (10 %)	
Dry weight, mean ± SD	72 ± 19	83 ± 23	−0.5	72 ± 20	−0.0
Primary insurer, n (%)					
Medicare	75 (35 %)	134,267 (58 %)	0.5	376 (35 %)	0.1
Medicaid	27 (12 %)	27,062 (12 %)		124 (11 %)	
Other	96 (44 %)	52,829 (23 %)		506 (47 %)	
Unknown	19 (9 %)	16,510 (7 %)		79 (7 %)	
Dual eligibility, n (%)	28 (13 %)	38,049 (17 %)	−0.1	139 (13 %)	0.0
Access type, n (%)					
Fistula/graft	33 (15 %)	64,580 (28 %)	0.3	180 (17 %)	0.0
Catheter	178 (82 %)	159,418 (69 %)		876 (81 %)	
PD	6 (3 %)	6670 (3 %)		29 (3 %)	
Diabetes, n (%)	23 (11 %)	90,661 (39 %)	−0.7	113 (10 %)	0.0
Hypertension, n (%)	101 (47 %)	118,361 (51 %)	−0.1	481 (44 %)	0.0
Coronary artery disease, n (%)	11 (5 %)	22,358 (10 %)	−0.2	44 (4 %)	0.0
Congestive heart failure, n (%)	20 (9 %)	41,999 (18 %)	−0.3	89 (8 %)	0.0
Cerebrovascular disease, n (%)	7 (3 %)	10,157 (4 %)	−0.1	34 (3 %)	0.0
Peripheral arterial disease, n (%)	4 (2 %)	15,866 (7 %)	−0.2	17 (2 %)	0.0
Charlson comorbidity index, n (%)					
2	104 (48 %)	19,284 (8 %)	−1.3	525 (48 %)	0.1
3	37 (17 %)	17,658 (8 %)		179 (17 %)	
4	32 (15 %)	36,889 (16 %)		180 (17 %)	
5	22 (10 %)	48,370 (21 %)		116 (11 %)	
6	15 (7 %)	51,966 (23 %)		57 (5 %)	
7	4 (2 %)	33,415 (14 %)		19 (2 %)	
8+	3 (1 %)	23,086 (10 %)		9 (1 %)	

*HUS* hemolytic uremic syndrome, *PD* peritoneal dialysis, *SD* standard deviation, *Std diff* standard difference

HUS patients and control patients contributed 315 and 1,850 years at risk, respectively. During this time, 39 and 204 deaths were observed corresponding to crude mortality rates of 12.4 deaths per 100 patient-years for HUS patients and 11.0 deaths per 100 patient-years for control patients. Accounting for the matched design, the hazard ratio [95 % confidence interval (CI)] for death for HUS patients versus control patients was 1.1 (0.8–1.7; p = 0.5) (Table 2).

**Table 2:** Survival comparison between hemolytic uremic syndrome patients and matched control patients

	N	Deaths	Cumulative at-risk time (years)	Mortality rate per 100 patient (years)	HR (95 % CI)
HUS patients	217	39	315.4	12.4	1.1 (0.8–1.7) p = 0.5
Control patients	1,085	204	1,850.2	11.0	

*CI* confidence interval, *HR* hazard ratio, *HUS* hemolytic uremic syndrome

**Fig. 2:**
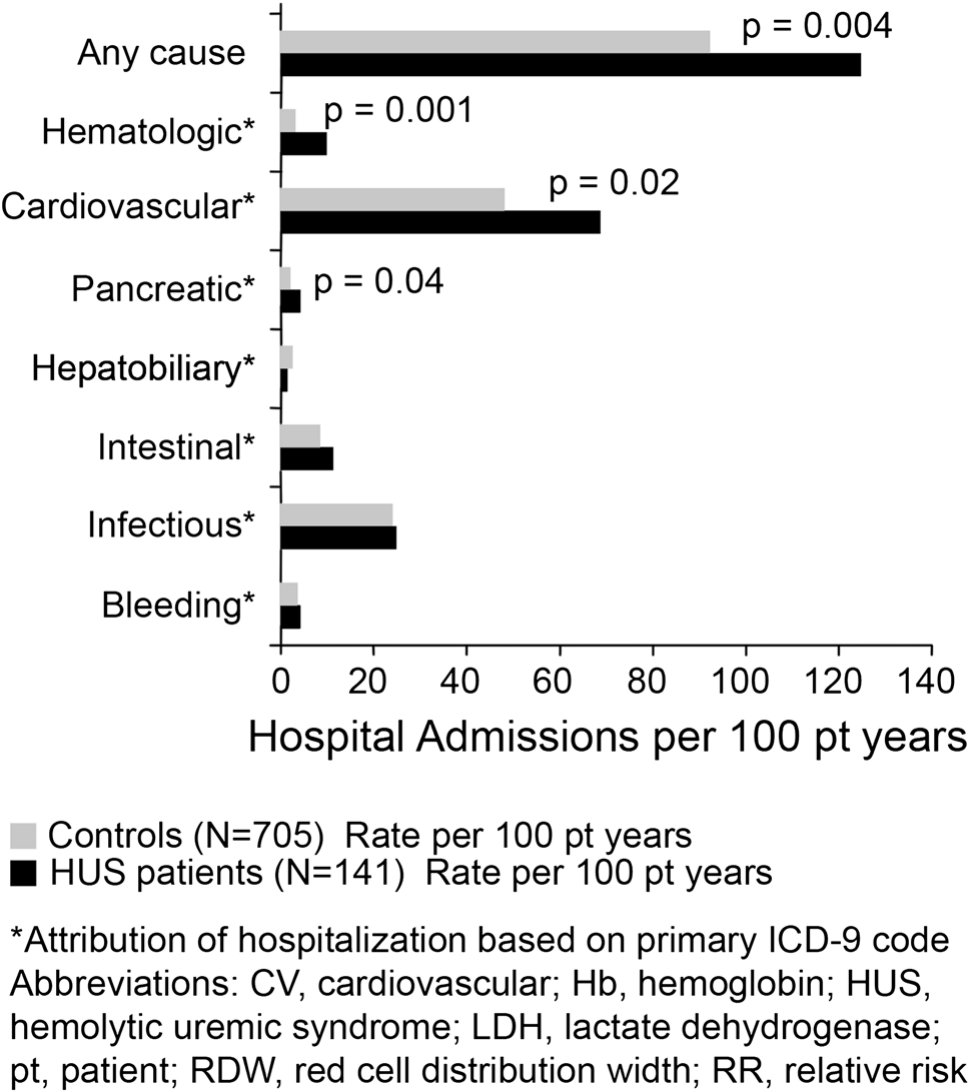
Hospital admission rates for dialysis patients with end-stage renal disease ascribed to hemolytic uremic syndrome compared to dialysis patients with end-stage renal disease ascribed to other causes

For hospitalizations, cumulative at-risk time was 141.2 patient-years for HUS patients and 779.1 patient-years for matched control patients (Table 3). A total of 176 hospitalizations were observed among HUS patients and 719 among control patients: hospitalization rates were 124.7 and 92.3 hospitalizations per 100 patient-years, respectively (Fig. 2). Incidence rate ratio (IRR) for hospitalization among HUS patients versus control patients was 2.3 (1.3–4.1; p = 0.004).

**Table 3:** Hospitalization rate comparison between hemolytic uremic syndrome patients and matched control patients

Hospitalization	HUS patients (N = 141)		Control patients (N = 705)		IRR (95 % CI)	p
	Hospital admissions	Rate per 100 pt years	Hospital admissions	Rate per 100 pt years		
Any cause	176	124.7	719	92.3	2.3 (1.3–4.1)	0.004
Hematologic^a^	14	9.9	25	3.2	5.6 (1.9–15.9)	0.001
Cardiovascular^a^						
Overall	97	68.7	375	48.1	2.1 (1.1–4.0)	0.02
Coronary arterial	4	2.8	21	2.7	1.3 (0.1–12.6)	0.8
Cerebrovascular	6	4.2	30	3.9	0.7 (0.1–4.6)	0.7
Peripheral arterial	0	0	8	1.0	–	–
VTE	0	0	4	0.5	–	–
Hypertensive crisis	7	5.0	15	1.9	5.6 (0.5–57.9)	0.2
Pulmonary HTN	0	0	0	0	–	–
Other CV	80	56.7	297	38.1	2.4 (1.3–4.4)	0.05
Pancreatic^a^	6	4.2	16	2.1	7.9 (1.1–59.8)	0.04
Hepatobiliary^a^	2	1.4	20	2.6	0.8 (0.0–17.8)	0.9
Intestinal^a^	16	11.3	66	8.5	1.8 (0.6–5.2)	0.3
Infectious^a^	35	24.8	187	24.0	1.2 (0.6–2.3)	0.6
Bleeding^a^	6	4.2	28	3.6	1.0 (0.3–3.3)	1.0

*CI* confidence interval, *CV* cardiovascular, *HTN* hypertension, *HUS* hemolytic uremic syndrome, *IRR* incidence rate ratio, *pt* patient, *VTE* venothromboembolism

^a^Attribution of hospitalization based on primary ICD-9 code

When hospitalizations were considered by primary cause, it was observed that the rate of hospitalization for hematological causes [IRR 5.6 (1.9–15.9); p = 0.001], cardiovascular causes [IRR 2.1 (1.1–4.0); p = 0.02], and pancreatic causes [IRR 7.9 (1.1–59.8); p = 0.04] were greater among HUS patients versus matched controls.

Considered longitudinally, HUS patients compared to control patients had higher mean lactate dehydrogenase levels (215.9 versus 193.9 U/L, p < 0.001), lower platelet levels (240.1 versus 248.1 per µL, p < 0.001), lower mean hemoglobin levels (11.1 versus 11.3 g/dL, p < 0.001), and higher RDW (15.6 versus 15.3 %, p < 0.001) (Table 4).

**Table 4:** Longitudinal laboratory values for thrombotic microangiopathy-related variables between hemolytic uremic syndrome patients and control patients

Variables	HUS patients		Control patients		p
	Pt months	Value	Pt months	Value	
Average lactate dehydrogenase (U/L), mean ± SD	2,655	215.9 ± 114.3	16,665	193.9 ± 65.9	<0.001
Average platelet count (no./µL), mean ± SD	2,594	240.1 ± 115.931	16,336	248.1 ± 89.0	<0.001
Average hemoglobin (g/dL), mean ± SD	2,754	11.1 ± 1.4	17,250	11.3 ± 1.3	<0.001
Average RDW (%), mean ± SD	2,590	15.6 ± 2.1	16,338	15.3 ± 1.9	<0.001
Lactate dehydrogenase spikes, n (%)	2,367	77 (3.3 %)	15,356	373 (2.4 %)	0.02

*HUS* hemolytic uremic syndrome, *Pt* patient, *RDW* red cell distribution width, *SD* standard deviation

**Fig. 3:**
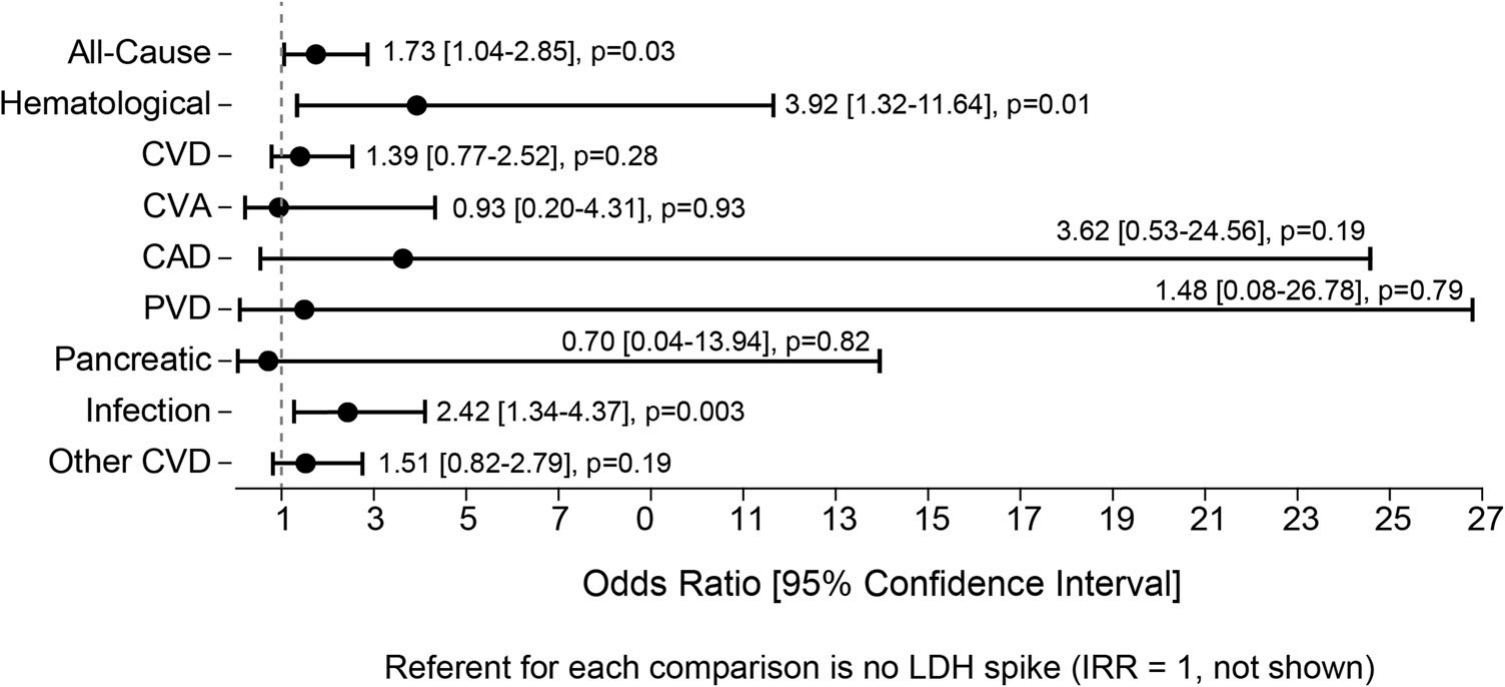
Temporal associations between lactate dehydrogenase (LDH) spikes (LDH > 100 compared to the mean of the prior 2 months) and hospitalization events were examined in the hemolytic uremic syndrome patients and controls. Significant associations were found for hospitalization of all-causes [1.73 (1.04–2.85; p = 0.03)], hematological causes [3.92 (1.32–11.6; p = 0.01)], and infection [2.42 (1.34–4.37; p = 0.003)]

Lactate dehydrogenase spikes were observed in 77 of 2,367 patient months (3.3 %) among HUS patients and 373 of 15,356 patient months (2.4 %) among matched controls: odds ratio (OR) (95 % CI) = 1.42 (1.05–1.91); p = 0.02. In the pooled population, lactate dehydrogenase spikes were associated with a greater risk of subsequent hospitalization overall [OR (95 % CI): 1.73 (1.04–2.85); p = 0.03], hospitalization for hematological causes [OR (95 % CI): 3.92 (1.32–11.64); p = 0.01] and for infection-related causes [OR (95 % CI): 2.42 (1.34–4.37); p = 0.003]; no significant associations were seen for other types of hospitalization (Fig. 3).

## Discussion

Despite the ongoing belief that the presence of HUS becomes moot once ESRD is reached, this study provides supporting evidence that TMA continues to manifest after dialysis is started in patients with HUS-ascribed ESRD. Among these patients, we found higher hospitalization rates, particularly for cardiovascular, hematologic, and pancreatic causes, which are the types of hospitalizations associated with TMA. We also found other evidence of ongoing TMA in the HUS-ascribed ESRD patients, including consistently higher lactate dehydrogenase and RDW levels, as well as lower platelet and hemoglobin levels, which may reflect a chronic ongoing low-grade disease process.

Earlier studies have found high TMA recurrence rates among HUS patients, Shumak et al. [15] found that one third of their HUS patients relapsed within a year, and Hayward et al. [16] found a relapse rate of 21.1 % in the first year following plasma treatment for HUS. More recent studies have found lower rates of TMA recurrence [5, 11].

A further indication of ongoing TMA was the increased frequency of lactate dehydrogenase spikes among HUS patients; this is suggestive of superimposed periods of disease acceleration. It was notable that hospitalization rates for TMA-related causes (cardiovascular, hematologic, and infections) were more common at the time of the lactate dehydrogenase spike versus other times.

This is the first study to compare HUS and non-HUS-ascribed ESRD patients in relation to hospitalization. Prior research has examined the morbidity and mortality of typical HUS versus atypical HUS patients. One study found that patients with atypical HUS are hospitalized more than twice as long during acute episodes compared to those with typical HUS [5].

The current study found no material difference in survival between HUS patients and control patients, a finding supported by an Australian study that found HUS-ascribed ESRD patients had comparable patient survival while on dialysis [17].

On balance, there is an important burden of HUS among patients who have already manifested ESRD vis-à-vis hospitalizations, in particular hospitalizations for cardiovascular, anemia, and pancreatic causes, which are associated with ongoing TMA activity. The current study provides additional evidence to support the hypothesis that TMA persists among HUS patients during ESRD as evidenced by increased hospitalizations and increased TMA laboratory findings (lower hemoglobin and platelets, higher lactate dehydrogenase and RDW levels, and more lactate dehydrogenase spikes).

One important limitation of this study is the inability to distinguish between atypical and diarrheal-associated HUS. Research has shown that atypical HUS patients have poorer outcomes than those with diarrheal-associated HUS [1, 18, 19]. Because the latter type of patients are generally healthier than atypical HUS patients, our findings likely underestimate the true burden of atypical HUS. Because of this, studies are needed to test whether directed treatment of atypical HUS reduces hospitalization rates.

Because this study is retrospective and observational, there may be some confounding. Propensity score matching was utilized to help minimize confounding.

In conclusion, comparing HUS patients to control patients—both with ESRD—HUS patients had significantly greater rates of hospitalization, particularly for cardiovascular, hematologic, and pancreatic disease. In addition, HUS patients exhibited laboratory evidence consistent with ongoing TMA, which is consistent with the idea that treatment for HUS may reduce morbidity and hospitalization rates. Efforts should be directed at identifying patients with ESRD ascribed to HUS to include HUS treatment to mitigate TMA events.
